# Supplementary material for: Requirement of Zebrafish Adcy3a and Adcy5 in Melanosome Dispersion and Melanocyte Stripe Formation
Source: Int J Mol Sci. 2022 Nov 16;23(22):14182. doi: 10.3390/ijms232214182 (PMC9693263; doi:10.3390/ijms232214182)
Supplement: Supplementary file 1 [file ijms-23-14182-s001.zip › ijms-1989236-supplementary.pdf]

Supplementary Materials

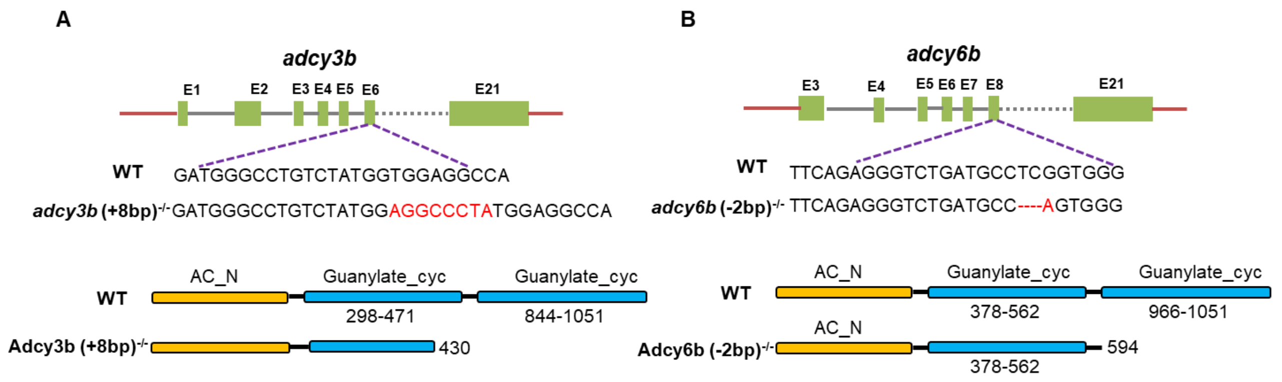

Figure S1. Knock-out of *adcy3b* and *adcy6b* by CRISPR/Cas9.
(**A**,**B**) The sgRNA target was designed in the sixth exon of *adcy3b* (**A**) and the eighth exon of *adcy6b* (**B**). Schematic representations of the nucleotide sequences showed an 8-bp insertion in *adcy3b*$^{-/-}$ and a 2-bp deletion and a 1-bp substitution in *adcy6b*$^{-/-}$. Deletions were shown as red dashes. The inserted bases and substitutive bases are marked in red. Predicted domain structure of Adcy3b and Adcy6b from wild-type and the mutants.

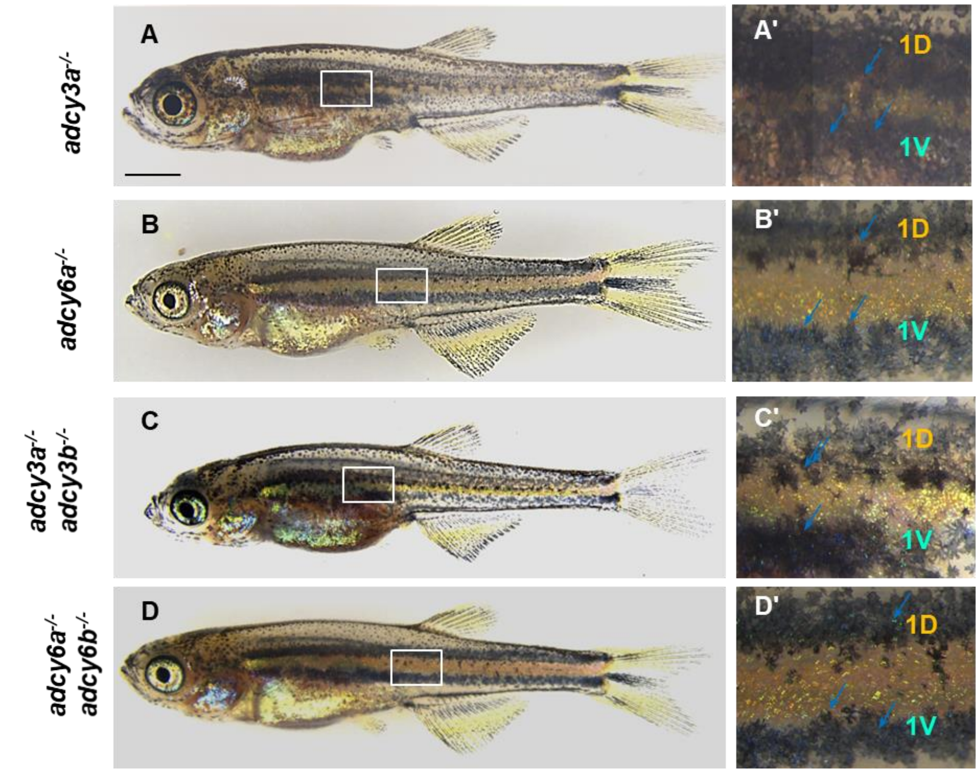

Figure S2. Analyses of melanin pattern in *adcy* mutants at later metamorphosis.
(**A–D**) *adcy3a*$^{-/-}$(**A**) and *adcy6a*$^{-/-}$ (**B**) single mutants, and double mutants *adcy3a*$^{-/-}$*adcy3b*$^{-/-}$ (**C**) and *adcy6a*$^{-/-}$*adcy6b*$^{-/-}$ (**D**) developed normal metamorphic melanocyte stripes, including dorsal (1D) and ventral primary melanocyte stripes (1V). The corolla distribution of melanosomes in melanocytes was visualized in the trunk skin (**A**′, **B**′, **C**′, **D**′). (**A**′**–D**′) are the magnified images of boxes in panels (**A–D**), respectively. The blue arrows indicate the corolla pattern of melanosomes. Scale bar = 1000 um in (**A–D**).

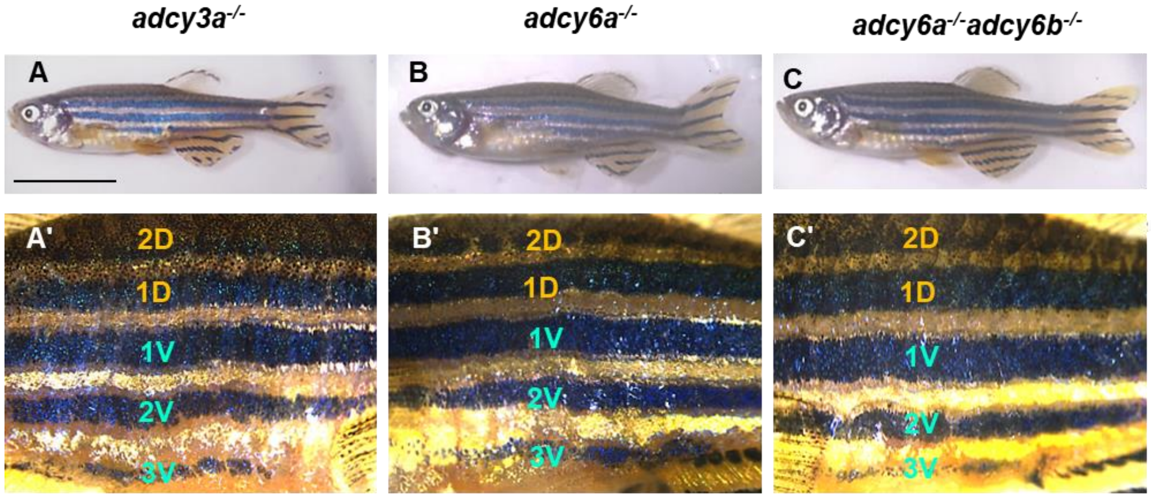

Figure S3. Analyses of melanocyte stripes in *adcy* mutants at the adult stage.
(**A–C**) Adults of *adcy3a*$^{-/-}$ (**A**), *adcy6a*$^{-/-}$ (**B**), and *adcy6a*$^{-/-}$*adcy6b*$^{-/}$ mutants (**C**) showed normal melanocyte stripe patterns, including three ventral black stripes 1V, 2V, and 3V, and two dorsal stripes. 1D: dorsal primary dark stripe; 1V: ventral primary dark stripe; 2D: dorsal secondary dark stripe; 2V ventral secondary dark stripe; 3V: the third ventral stripe. A′-C′ are the partially magnified images of (**A–C**), respectively. scale bar = 10 mm in (**A–C**).

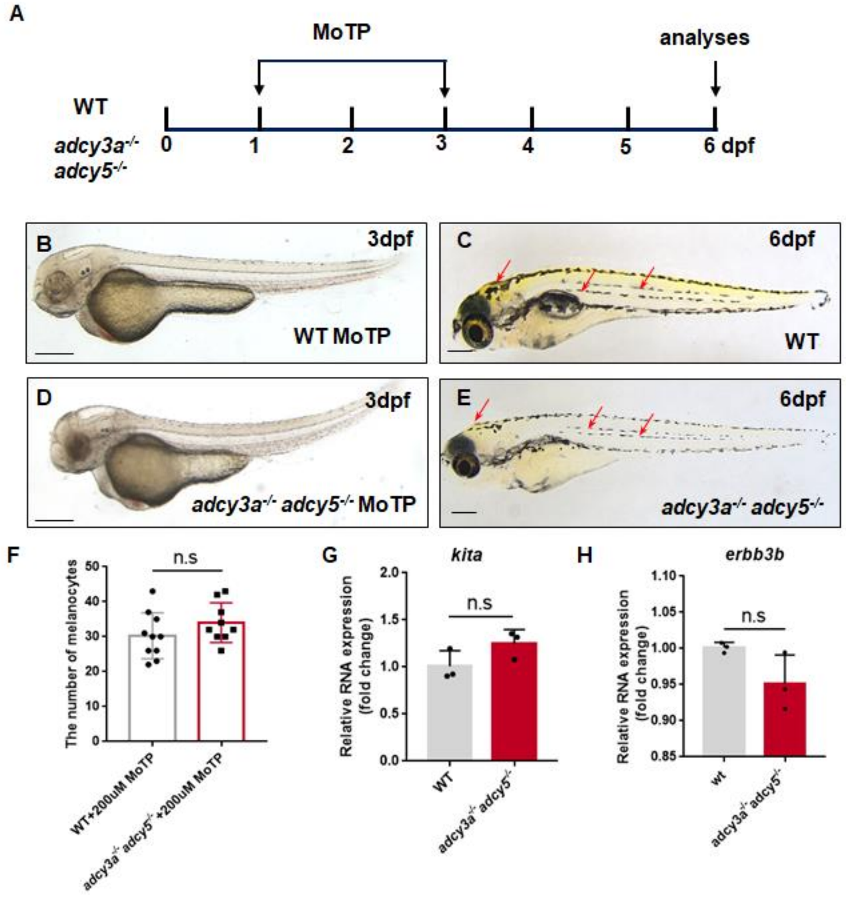

Figure S4. ***adcy3a*** **and** ***adcy5*** **double mutations do not affect the establishment of MSC.**
(**A**) Schematic diagram for experimental procedures with MoTP treatment. Zebrafish embryos were treated with MoTP from 1 to 3 dpf. After the washout of MoTP, the regenerated melanocyte number is quantified at 6 dpf. (**B–E**) No melanocytes were observed when WT embryos (**B**) or *adcy3a*$^{-/-}$*adcy5*$^{-/-}$ mutants (**D**) were incubated in MoTP solution from 1 to 3 dpf. The regenerated melanocytes were observed in the head and the

trunk at 6 dpf in WT embryos (**C**) and *adcy3a*$^{-/-}$*adcy5*$^{-/-}$ mutants (**E**). The red arrows indicate some regenerated melanocytes. Scale Bar = 250 um. (**F**) Statistical analyses showed that there was no significant difference in the mean number of regenerated melanocytes in the lateral stripe between WT larvae and *adcy3a*$^{-/-}$*adcy5*$^{-/-}$ mutants (Student's *t*-test, n.s., not significant, $p > 0.05$). (**H**) qPCR analyses showed mRNA levels of *kita* and *erbb3b* in WT embryos and *adcy3a*$^{-/-}$*adcy5*$^{-/-}$ double mutants (Student's *t*-test, n.s., not significant, $p > 0.05$).
